# Postpartum Spontaneous Subcapsular Hepatic Hematoma Related to Preeclampsia

**DOI:** 10.1155/2014/417406

**Published:** 2014-08-17

**Authors:** Dimitrios Anyfantakis, Miltiades Kastanakis, Georgios Fragiadakis, Paraskevi Karona, Nikolaos Katsougris, Emmanouil Bobolakis

**Affiliations:** ^1^Primary Health Care Centre of Kissamos, Loulakaki 13, Lentariana, 73134 Chania, Crete, Greece; ^2^First Department of Surgery, Saint George General Hospital of Chania, 73100 Crete, Greece

## Abstract

Subcapsular hematoma of the liver represents an unusual clinical phenomenon in the pregnancy and postpartum period with serious complications in terms of fetal and maternal mortality. Here we report a case of a 32-year-old primiparous female at 36 weeks of gestation, admitted to a maternity ward of a private clinic for preeclampsia. The woman underwent an emergency caesarean section with the extraction of an alive foetus. A few hours after delivery, she was transferred to the emergency department of our institution complaining of severe epigastric pain. Diagnostic work-up was suggestive of a subcapsular right lob hepatic hematoma which was successfully managed conservatively. Timely diagnosis is necessary for the prevention of life-threatening events in mother and fetus. For this reason acute care physicians have to be vigilant of the condition and consider this in the differential diagnosis of epigastric pain during pregnancy and postpartum.

## 1. Introduction

Liver function abnormalities during pregnancy are considered unusual and can range from a mild elevation of plasma liver enzyme levels to severe functional impairment with poor maternal and neonatal outcomes (in approximately 3% to 5% of deliveries) [1]. Spontaneous subcapsular hepatic hematoma represents a life threatening complication which is often associated with preeclampsia and HELLP (hemolysis, elevated liver enzymes, and low platelet count) syndrome [2].

In this paper we describe a case of spontaneous hepatic hematoma developed in a 32-year-old prim-gravid woman a few hours after an emergency cesarean section due to preeclampsia.

## 2. Case Report

A 32-year-old prim-gravid Greek female was transferred from the maternity ward of a private clinic to the emergency department of the Saint George General Hospital of Chania, Crete, Greece, complaining of acute abdominal pain located on the right upper quadrant radiated to the right shoulder. Eight hours before, the woman underwent an emergency caesarian section at the 36th week of gestation due to preeclampsia and fetal distress. Before caesarian section, blood pressure control was obtained with the use of nifedipine in a dosage of 10 mg per os twice, every 30 minutes. Intravenous magnesium sulfate was also administrated for the prevention of eclamptic seizures. A healthy female infant weighing 3.200 g was delivered.

**Figure 1:**
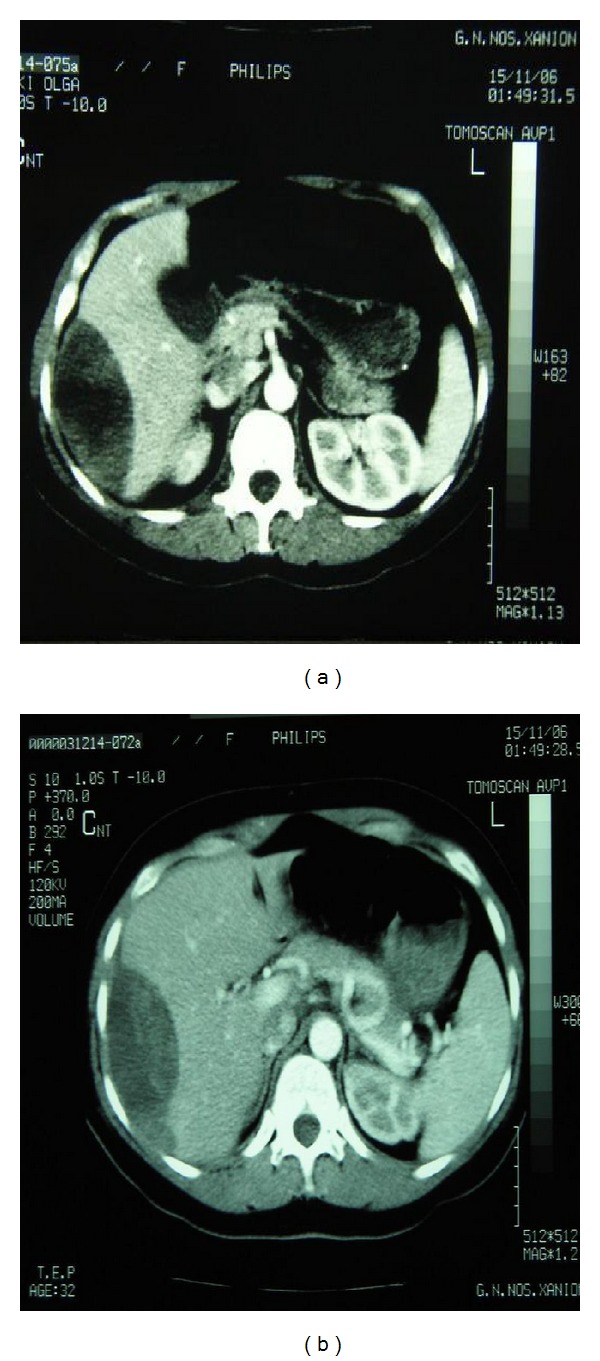
Abdominal computed tomography demonstrating the existence of a large subcapsular hepatic hematoma.

Her vital signs on admission to our institution were all within normal limits except of an elevated level of blood pressure (185/95 mmHg). Initial laboratory work-up showed the following: hematocrit (HCt) 29.3% (normal range 35–47%), hemoglobin (Hb) 9 gr/dL (normal range 11.5–15.5 gr/dL), platelet count (PLT) 185000/*μ*L (normal range 150000–450000/*μ*L), white blood cell count (WBC) 12200 cells/mm^3^ (normal range 4000–11000 cells/mm^3^), aspartate-aminotransferase 67 U/L (normal range 0–38 U/L), alanine aminotransferase 111 U/L (normal range 4–36 U/L), and lactic dehydrogenase (LDH) 682 UL (normal range 240–480 UL). Coagulation profile included examination of partial thromboplastin time (PTT) and of activated partial thromboplastin time (aPTT) which were within normal ranges. Urine analysis demonstrated proteinuria. Physical examination disclosed a light tenderness of the right upper abdominal quadrant. Abdominal ultrasound disclosed subcapsular fluid collection in the right hepatic lobe. Further imaging included computed tomography (CT) of the abdomen which confirmed the diagnosis demonstrating a large well-circumscribed subcapsular liver hematoma with intact capsula (15 × 10 × 14 cm) in the right hepatic lobe (Figures 1(a) and 1(b)).

**Figure 2:**
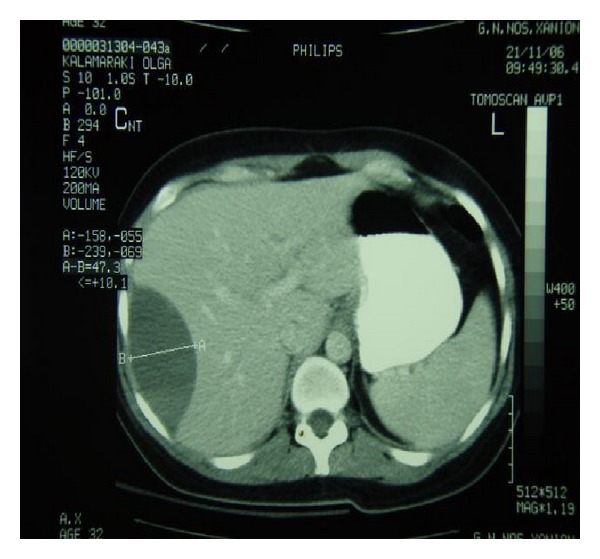
Follow-up CT scan showing decrease of the dimensions of the hematoma.

**Figure 3:**
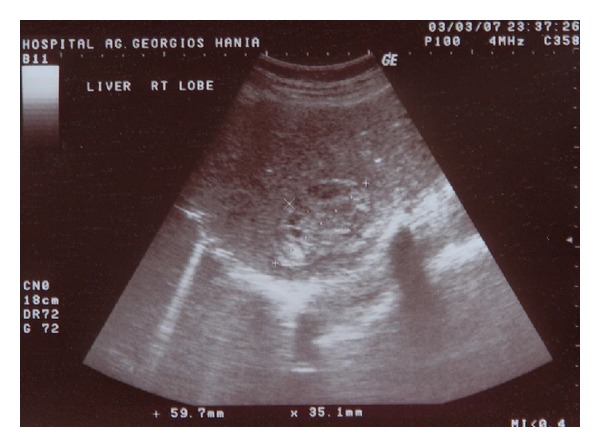
Abdominal ultrasound performed 3 months later reveals further reduction of the hematoma's dimensions.

**Figure 4:**
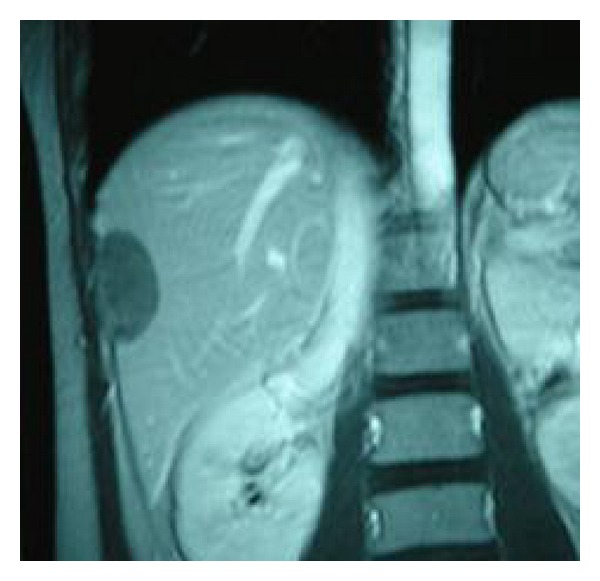
MRI showing diminished size of the hematoma.

Since no active bleeding was identified at that time, the patient was managed conservatively with fluid infusions, antihypertensive medication, and antibiotherapy. Her clinical status as well as hematocrit and liver enzymes levels was improved significantly during the following days. A follow-up CT scan in the 15th hospital day disclosed a significant decrease in the dimensions of the hematoma (Figure 2). The patient was discharged home on the 19th day of hospitalization following an uneventful clinical course. An ultrasound and magnetic resonance imaging (MRI) abdominal scan performed 3 months and 5 months later, respectively, showed that hematoma had further reduced to 5.9 × 3.5 cm (Figure 3) and 2 × 3 × 4.5 cm (Figure 4). Nine-month postpartum abdominal ultrasound showed no residual hematoma.

## 3. Discussion

Subcapsular hepatic hematoma is the accumulation of blood between the capsule of Glisson and the liver parenchyma [3]. It was first described by Abercombie in 1844 [4]. Remarkably, the etiopathogenesis still remains unclear [5]. An interesting hypothesis is based on the formation of fibrin thrombus within the hepatic arteries and sinusoid capillaries which in turn leads to periportal necrosis, intrahepatic hemorrhage, and finally subcapsular hematoma [5]. In the vast majority of cases (75% of the patients) right hepatic lobe is more frequently affected [6].

The HELLP syndrome occurs in approximately 0.5%–0.9% of all pregnancies with preponderance in white and multiparous females having a delayed diagnosis of preeclampsia [7, 8]. In the majority of cases (approximately 7 out of 10) the syndrome develops before delivery [7]. Disseminated intravascular coagulation represents one frequent complication of the HELLP syndrome with a reported occurrence of 15% to 20% [8].

Clinical symptoms and signs are nonspecific and range from epigastric or right upper quadrant abdominal pain with shoulder irradiation to nausea, vomiting, and abdominal distension. In case of rupture in the abdomen, signs of hemodynamic compromise may develop [6]. Abdominal ultrasound represents a useful first choice noninvasive tool for diagnosis and evaluation [5]. CT and MRI could be used in order to elucidate the diagnosis in ambiguous cases [5]. Management in hemodynamically stable patients is mostly conservative and includes intensive fluid replacement as well as blood and fresh-frozen plasma transfusions [5]. In some cases percutaneous transcatheter hepatic artery embolization could be a useful alternative procedure that could effectively control haemorrhage [5]. Hemodynamic instability requires urgent surgery [5]. Bleeding surfaces are packed with collagen fleece and perihepatic space is drained. Liver transplantation in specialized tertiary care centers is reserved in some cases of acute hepatic failure due to unmanageable liver bleeding [5, 9]. Abdominal ultrasound, CT, and MRI are useful imaging tools included in the postpartum follow-up until the resolution of the hematoma [9]. In our case clinical symptoms in combination with the impaired liver function tests raised the diagnostic suspicion. Since hemodynamic status of our patient was not compromised we followed a successful noninvasive conservative management. In the same direction in a 10-year retrospective study of 10 patients with subcapsular liver hematoma, 3 of them were successfully managed conservatively [4].

Surgeons as well as obstetricians have to cultivate a high level of vigilance for rare clinical phenomena such as subcapsular hepatic hematomas. Early diagnosis could decrease morbidity and mortality burden for both mother and fetus.
